# Social Determinants and Self-Care for Making Good Treatment Decisions and Treatment Participation in Older Adults: A Cross-Sectional Survey Study

**DOI:** 10.3390/nursrep12010020

**Published:** 2022-03-10

**Authors:** Udoka Okpalauwaekwe, Chih-Ying Li, Huey-Ming Tzeng

**Affiliations:** 1 College of Medicine, University of Saskatchewan, Saskatoon, SK S7N 2Z4, Canada; udokaokpala.uo@usask.ca; 2 Department of Occupational Therapy, School of Health Professions, The University of Texas Medical Branch, Galveston, TX 77555, USA; chili@utmb.edu; 3 School of Nursing, The University of Texas Medical Branch, 301 University Boulevard, Galveston, TX 77555, USA

**Keywords:** person-centered care, patient participation, self-care, patient engagement, shared decision-making, informed care planning

## Abstract

**Background:** Community-dwelling adults who can perform self-care behaviors related to making treatment decisions and participating in treatment have been found to use less emergency care. In this exploratory study, we examined the relationships in older adults between five social determinants (urban/rural residence, sex, age, marital status, and education) and the perceived importance, desirability, and ability to perform 11 self-care behaviors related to making good treatment decisions and participating in treatment. **Methods:** This cross-sectional study surveyed 123 community-dwelling older adults living in the southern United States in 2015–2016. All participants were 65 years or older. Data were collected using the Patient Action Inventory for Self-Care and analyzed using descriptive, univariate, and multivariate logistic regression analyses. **Results:** The social determinants (identified as barriers) of self-care behaviors related to making good treatment decisions and participating in treatment were: having less than a high school education, being 75 years or older, and being separated from a spouse. Sex and residence were found to be neither barriers nor facilitators. **Conclusions:** Our findings suggest that, in older adults, attending to the needs related to health literacy education and improving social support might increase self-care behaviors related to making good treatment decisions and participating in treatment. Future research will compare the differences across diverse populations to validate our study findings.

## 1. Introduction

The U.S. Agency for Healthcare Research and Quality recommends that the effective engagement of patients in their self-care improves health outcomes, patient satisfaction, and overall quality of life [1]. This patient-centered approach to healthcare advocates for a patient’s involvement in making good treatment decisions while participating in their treatment through shared decision-making [1]. Shared decision-making is the process in which a healthcare treatment choice is made jointly between the patient (and/or family member) and one or more healthcare professionals [2]. Power imbalances in patient–clinician relationships and the perception of patient participation acceptability on the part of clinicians are often cited as barriers to engaging patients in shared decision-making [3,4]. Although decision aids have been shown to help support patients in shared decision-making processes, most older adult patients need both the knowledge and the power to actively engage in decision-making about their treatment plans [3]. Hence, recent studies have recommended adding patient engagement and self-care activities to the process [5,6,7,8].

Events brought about by the ongoing global COVID-19 pandemic have no doubt informed the need for a patient-centered approach to promoting self-care for older adults. Health challenges and disparities have clearly been amplified by the advent of COVID-19, especially in the more aged population. The U.S. Centers for Disease Control and Prevention reported that adults 65 years and older are more likely than those of other age groups to die or develop a severe illness leading to hospitalization if they contract the coronavirus [9], which resulted in recommendations for older adults to stay at home and avoid close contact with others. Already, several studies have reported on the compounding health conditions among older adults—anxiety, depression, loneliness, increasing comorbidities, and the worsening of existing chronic diseases—brought about as they were considered the most vulnerable to COVID-19 [10,11,12,13,14]. These studies also implied that older adults desired a sense of control and preparedness to make medical decisions involving their care [10,11,15,16].

The World Health Organization defines self-care as “the ability of individuals, families and communities to promote health, prevent disease, maintain health, and to cope with illness and disability with or without the support of a healthcare provider” [17]. Assessing the ability of older adults to handle self-care is a common barrier to engaging older adults in making good treatment decisions or participating in their care [2]. The notion that older adults are frail and vulnerable tends to challenge their active involvement in self-care and often relegates their care to nursing homes designed to provide more attention and care support. Contrarily, a few studies have shown that most older adults above the age of 65 consider themselves active and in better physical and psychological health than labels often suggest [8,10,18]. A recent survey of more than 1000 older adults in Western Canada exploring the question “What suggestions can you make to engage someone in their health and healthcare?” indicated that older adults wanted to be engaged as partners in their care in ways that enhance feasible healthcare access and autonomy in making decisions [8]. Another recent U.S. study [19] found that community-dwelling adults (53.7% of whom were 65 years and older) who reported being able to perform the self-care behaviors of knowing about any interactions between their old and new treatments, talking with their providers when stopping treatment, and tracking their symptoms and health measures, were less likely to have visited the emergency department in the preceding three months. Yet, how the demographic social determinants of older adults affected their ability to perform self-care behaviors related to making good treatment decisions and participating in their treatment was unclear. A literature search for peer-reviewed journal articles showed that, compared with other counterparts, older adult men, married individuals, and individuals with a more advanced education were more likely to participate in shared decision-making self-care practices actively [3,20,21,22]. Advancing age was demonstrated in other studies to be a facilitator [23] or a barrier [24,25,26] to performing shared decision-making. Geographic location and residential setting were not associated with older adults’ self-care capacity to participate in shared decision-making or treatment [3,20,21,22].

### Study Rationale

To be able to develop practical solutions addressing the unique needs of older adults, we aimed to improve our understanding of the demographic, social determinants associated with their treatment decision-making and participation in treatment. In this exploratory study, we examined the relationships between five demographic variables of older adults (urban or rural residence, sex, age, marital status, and education level) and the perceived importance, desirability, and ability to perform 11 self-care behaviors related to making good treatment decisions and participating in their treatment.

## 2. Methods

### 2.1. Study Design

This study used data from a cross-sectional survey of community-dwelling adults living in the southern United States in 2015–2016. The project was approved by the Tennessee Technological University and the University of Saskatchewan institutional review boards. This manuscript complies with STROBE guidelines (https://www.equator-network.org/reporting-guidelines/strobe/, accessed on 7 March 2022).

### 2.2. Study Participants

A detailed description of the methods used in the study has been published [19,27]. Convenience sampling was used to recruit healthy community-dwelling adult participants 18 years and older living in the region. Eight seniors’ centers and the student health service at Tennessee Technological University were surveyed. The targeted sample size was 250. Responses from participants 65 years and older (*N* = 123) were used for the present study.

### 2.3. Data Collection Instruments

The self-administered survey included two tools:

- The Patient Action Inventory for Self-Care
  This tool was developed and validated by Tzeng and Pierson [27] based on the Engagement Behavior Framework developed by the Center for Advancing Health [28]. It encompasses 52 patient engagement behaviors grouped into 10 categories. The Cronbach alpha for the tool as a whole was 0.968 [27].
  For this study, we focused on 2 of the 10 categories: “making good treatment decisions” and “participating in treatment.” Participants were asked to select “yes” or “no” for each behavior statement from three perspectives: Is this important to you? Do you want to do this? Are you able to do this?
  Participant responses related to the 11 self-care behaviors (outcome variables) from among the 52 behaviors inventoried were analyzed: Seeking more than one expert opinion for the treatment of illness when needed; Asking about the good and bad outcomes of suggested treatments; Working with your provider(s) on your treatment plan; Knowing side effects before starting new treatments; Knowing how old and new treatments interact; Filling or refilling prescriptions on time; Keeping track of the outcomes of your treatments; Talking with your provider(s) when stopping your treatment; Maintaining all of your health devices; Discussing why tests are ordered before getting them done, and; Tracking your symptoms and health measures.
- Demographic Questionnaire
  This tool was used to gather information on age group (65 to <75 years, 75 to <85 years, and ≥85 years), sex (male and female), marital status (married, single, or separated), residential setting (urban or rural), and education level (<high school diploma; high school diploma; or ≥associate’s or bachelor’s degree). These demographic characteristics were then used as potential correlates.

### 2.4. Data Analysis

Data were processed using the IBM SPSS Statistics software application (version 25.0: IBM, Armonk, NY, USA). Partly completed surveys were included in the analysis. Missing data were left without imputation. Descriptive analyses were used to examine frequencies and means for the variables of interest. Univariate logistic regression analyses were performed to assess the contribution of each demographic trait individually. Categorical variables (whether respondents perceived each of the 11 self-care behaviors as important, desirable, and able to be performed) were coded as “yes” or “no”. Multivariate logistic regression analyses were performed using the default forced entry method to estimate adjusted odds ratios (ORs), 95% confidence intervals (CIs), and *p* values for the outcome variables. The sample size was computed statistically based on the work of Peduzzi et al. [29]. Although the sample size was sufficient for the univariate logistic regression analyses in the study, the multivariate logistic regression analyses were conducted as exploratory supplemental analyses. The level of significance (alpha) was set at 0.05 for two-sided statistical tests.

## 3. Results

Table 1 and Table 2 summarize the descriptive variables examined. The response rate was 82%. Of the 123 participants, most lived in a rural community (76, 61.8%), they were female (90, 73.3%), and they were 65 to less than 75 years of age (60, 48.8%). Table 3, Table 4 and Table 5 summarize the findings of the logistic regression analyses.

### 3.1. Univariate Logistic Regression

Of the univariate logistic regression models, six (one assessing importance, one assessing the desirability, and four assessing the ability to perform self-care behaviors related to making good treatment decisions and participating in treatment) contained at least one statistically significant regression coefficient value (*p* < 0.05; Table 3). The odds of responding “yes” to perceiving that it is important to perform the “seeking more than one expert opinion for the treatment of illness when needed” self-care behavior were lower for adults 85 years of age and older than for those 65 to less than 75 years of age (*p* = 0.036; OR: 0.177; 95% CI: 0.035 to 0.891). The odds of responding “yes” to having the desire to perform the “discussing why tests are ordered before getting them done” self-care behavior were lower for older adults separated from their spouses than for adults who were married (*p* = 0.021; OR: 0.057; 95% CI: 0.005 to 0.645).

The odds of reporting “yes” to being able to perform the “knowing side effects before starting new treatments” self-care behavior were higher for older adults with a high school diploma than for those without a high school diploma (*p* = 0.045; OR: 12.800; 95% CI: 1.060 to 154.578). The odds of reporting “yes” to being able to perform the “knowing of any interactions between old and new treatments” self-care behavior were lower for separated older adults than for those who were married (*p* = 0.026; OR: 0.114; 95% CI: 0.017 to 0.768). The odds of reporting “yes” to being able to perform the “knowing how old and new treatments interact” self-care behavior were lower for adults 75 to less than 85 years of age (*p* = 0.020; OR: 0.145; 95% CI: 0.029 to 0.739) and for those 85 years of age and older (*p* = 0.015; OR: 0.112; 95% CI: 0.019 to 0.649) than for those 65 to less than 75 years of age. The odds of reporting “yes” to being able to perform the “tracking your symptoms and health measures” self-care behavior were lower for adults 85 years of age and older than for those 65 to less than 75 years of age (*p* = 0.024; OR: 0.072; 95% CI: 0.007 to 0.704).

### 3.2. Supplemental Analyses

Two multivariate logistic regression models, which each included all five demographic variables of interest, contained at least one statistically significant regression coefficient value (Table 4 and Table 5).

## 4. Discussion

Our findings showed that having less than a high school education, being 75 years of age or older, and being separated from a spouse were potential barriers to making good treatment decisions and participating in one’s treatment. Sex and residential setting (rural versus urban) were neither barriers nor facilitators to performing self-care behaviors, which is consistent with the review findings in the literature.

Advancing age was a barrier to one of the three self-care behavior items related to making good treatment decisions (“seeking more than one expert opinion for the treatment of illness when needed”). A lower education level, separation from a spouse, and advancing age were potential barriers to at least one of the four self-care behaviors related to treatment participation (“knowing side effects before starting new treatments”, “knowing of any interactions between old and new treatments”, “discussing why tests are ordered before getting them done”, and “tracking your symptoms and health measures”). Those findings are consistent with results from previous studies that showed relationships between sociodemographic characteristics and engaging in shared decision-making in health settings [3,20,26].

The self-care needs for the older adult population are often summarized to include physical activity, stress management, and social and community support [4,11,19,30]. Those needs typically inform self-care practices that incorporate coping strategies, self-advocacy, the prioritization of self, legacy building, and activism [4,5,11,19,30]. Patients identified that, in order to facilitate shared decision-making, a multiple-consultation model that strategically allocates needed shared decision-making supports to various healthcare providers is needed. Patients viewed nurses as mediators in the shared decision-making process. As such, nurses could clarify treatment information, listen to patients’ preferences, and provide physicians with information about those preferences [3]. The barriers identified in our study could, therefore, be addressed by attitudinal changes at the patient, clinician or healthcare team, and healthcare organization levels.

Michie et al. [31] emphasized that patient engagement strategies involving the behavioral change model should consider a patient’s capacity, opportunity, and motivation to make the changes. Strategies to improve the self-care capacity in older adults might, therefore, consider a patient’s educational needs, family support, and self-efficacy levels, with the goal of improving their self-regulation [30]. Nurses and healthcare providers should consider the sequential relationship and the feedback loop of mind–emotion–behavioral readiness in older adults [30,31,32]. Strategies to assess this readiness can support each older adult’s unique needs in performing self-care related to making good treatment decisions and participating in treatment. Understanding which self-care behaviors are important to older adults and which ones they want to perform is the first step. Nurses and healthcare providers could then partner with older adults to address the self-care behaviors they desire to perform but lack the skills to perform.

In the course of the current COVID-19 pandemic, research continues to show that compared with adults in other age groups, adults 65 years and older, including those with pre-existing medical conditions, are more likely to develop a severe infection if they contract the coronavirus. A pertinent question to ponder is how, as healthcare practitioners and scientists, we can continue to provide meaningful and effective services to older adults. Public health agencies still advise engagement by older populations in self-care activities to limit stress-related adverse events and bolster overall health [11,12,13,14,16,33]; however, strategies should also consider the barriers of self-care capacity as they relate to making good treatment decisions and participating in treatment. To be more specific, the self-care behaviors associated with social determinants in older adults are “seeking more than one expert opinion for the treatment of illness when needed”, “knowing side effects before starting new treatments”, “knowing how old and new treatments interact”, and “tracking your symptoms and health measures”.

### Study Limitations and Future Research Directions

Because the data used in this study were collected in 2015–2016 from community-dwelling older adults residing in the southern United States, the findings might not be generalizable to patients living in other regions. Since completing the survey is voluntary, some participants may not answer every single question on the survey. Having missing values due to partially completed surveys is a study limitation. The relatively low response rate (49.2%) is another study limitation. Additionally, the Cronbach alpha for the tool as a whole was 0.968 [27]; the high Cronbach alpha for the survey tool suggested that there are limited variations on the participants’ responses across survey items, which would be another study limitation.

Future research is needed to compare the differences across diverse populations of older adults in the perceptions of self-care behaviors related to making good treatment decisions and participating in treatment. A future data collection must, therefore, include the health-related social determinants previously identified to affect individual and population health [34,35,36].

## 5. Conclusions

Our data showed that the potential barriers to performing self-care behaviors related to making good treatment decisions and participating in treatment were having less than a high school education, being 75 years of age or older, and being separated from a spouse. Our findings suggest that attending to health literacy education and improving social support for older adults might increase self-care behaviors related to making good treatment decisions and participating in treatment. Future research will evaluate the differences across diverse populations to validate our study findings.

## Figures and Tables

**Table 1:** Demographics of the 123 older community-dwelling adult participants.

Variable		Responses (*n* [%])
Residential site		
	Urban county	47 (38.2)
	Rural county	76 (61.8)
Sex		
	Female	90 (73.3)
	Male	23 (18.7)
	Missing	10 (8.1)
Age group		
	65 to <75 Years	60 (48.8)
	75 to <85 Years	44 (35.8)
	≥85 Years	19 (15.4)
Marital status		
	Married	48 (39.0)
	Single	49 (39.9)
	Separated	12 (9.8)
	Missing	14 (11.4)
Education		
	<High school diploma	18 (14.6)
	High school diploma	82 (66.7)
	≥Associate’s or bachelor’s degree	23 (18.7)
Ethnic group ^a^		
	White, non-Hispanic	111 (90.2)
	White, Hispanic	6 (4.9)
	Black or African American	1 (.8)
	American Indian or Alaska Native	5 (4.1)
	Asian	0 (0)
	Native Hawaiian or Pacific Islander	0 (0)
	Other	0 (0)

^a^ Not included in the analysis.

**Table 2:** Perspectives on self-care behaviors from the 123 older community-dwelling adult participants.

Behavior	Perception of Behavior (*n* [%])								
	Important to Perform			Desire to Perform			Able to Perform		
	No	Yes	Missing	No	Yes	Missing	No	Yes	Missing
Seeking more than one expert opinion for the treatment of illness when needed	13 (10.6)	103 (83.7)	7 (5.7)	14 (11.4)	78 (63.4)	31 (25.2)	11 (8.9)	88 (71.5)	24 (19.7)
Asking about the good and bad outcomes of suggested treatments	2 (1.6)	114 (92.7)	7 (5.7)	5 (4.1)	86 (69.9)	32 (26.0)	4 (3.3)	95 (77.2)	24 (19.5)
Working with your provider(s) on your treatment plan	2 (1.6)	112 (91.1)	9 (7.3)	4 (3.3)	86 (69.9)	33 (26.8)	3 (2.4)	95 (77.2)	25 (20.3)
Knowing side effects before starting new treatments	5 (4.1)	111 (90.2)	7 (5.7)	6 (4.9)	84 (68.3)	33 (26.8)	4 (3.3)	93 (75.6)	26 (21.1)
Knowing how old and knew treatments interact	10 (8.1)	103 (83.7)	10 (8.1)	8 (6.5)	80 (65.0)	35 (28.5)	15 (12.2)	80 (65.0)	28 (22.8)
Filling or refilling prescriptions on time	0 (0.0)	116 (94.3)	7 (5.7)	2 (1.6)	89 (72.4)	32 (26.0)	0 (0.0)	99 (80.5)	24 (19.5)
Keeping track of the outcomes of your treatments	1 (0.8)	113 (91.9)	9 (7.3)	6 (4.9)	84 (68.3)	33 (26.8)	3 (2.4)	94 (76.4)	26 (21.1)
Talking with your provider(s) when stopping your treatment	6 (4.9)	108 (87.8)	9 (7.3)	8 (6.5)	81 (65.9)	34 (27.6)	5 (4.1)	93 (75.6)	25 (20.3)
Maintaining all of your health devices	3 (2.4)	102 (82.9)	18 (14.6)	8 (6.5)	74 (60.2)	41 (33.3)	3 (2.4)	89 (72.4)	31 (25.2)
Discussing why tests are ordered before getting them done	3 (2.4)	111 (90.2)	9 (7.3)	8 (6.5)	84 (68.3)	31 (25.2)	3 (2.4)	96 (78.0)	24 (19.5)
Tracking your symptoms and health measures	5 (4.1)	105 (85.4)	13 (10.6)	8 (6.5)	80 (65.0)	35 (28.5)	7 (5.7)	87 (70.7)	29 (23.6)

**Table 3:** Univariate logistic regression predicting the likelihood of a “yes” response to a perception about performing a self-care behavior.

Behavior	Perception of Behavior	Comparators	β	SE	Wald	df	*p* Value ^a^	OR	95% CI
Seeking more than one expert opinion for the treatment of illness when needed	Important to perform	65 to <75 Years (reference)			4.581	2	0.101		
		75 to <85 Years	−1.145	0.739	2.398	1	0.121	0.318	0.075 to 1.355
		≥85 Years	−1.730	0.824	4.412	1	**0.036**	0.177	0.035 to 0.891
Discussing why tests are ordered before getting them done	Desire to perform	Married (reference)			6.203	2	0.045		
		Single	−1.157	1.180	0.962	1	0.327	0.314	0.031 to 3.174
		Separated	−2.862	1.236	5.359	1	**0.021**	0.057	0.005 to 0.645
Knowing side effects before starting new treatments	Able to perform	<High school (reference)			4.128	2	0.127		
		High school diploma	2.549	1.271	4.023	1	**0.045**	12.800	1.060 to 154.578
		≥Associate’s or bachelor’s degree	1.335	1.286	1.078	1	0.299	3.800	0.306 to 47.211
Knowing how old and new treatments interact	Able to perform	Married (reference)			4.979	2	0.083		
		Single	−.618	0.672	0.847	1	0.357	0.539	0.144 to 2.011
		Separated	−2.169	0.972	4.977	1	**0.026**	0.114	0.017 to 0.768
	Able to perform	65 to <75 Years (reference)			6.695	2	0.035		
		75 to <85 Years	−1.929	0.830	5.404	1	**0.020**	0.145	0.029 to 0.739
		≥85 Years	−2.193	0.898	5.960	1	**0.015**	0.112	0.019 to 0.649
Tracking your symptoms and health measures	Able to perform	65 to <75 Years (reference)			6.062	2	0.048		
		75 to <85 Years	−1.133	1.248	0.824	1	0.364	0.322	0.028 to 3.717
		≥85 Years	−2.628	1.162	5.119	1	**0.024**	0.072	0.007 to 0.704

^a^ Values in boldface type are statistically significant (<0.05). SE = standard error; df = degrees of freedom; OR = odds ratio; and CI = confidence interval.

**Table 4:** Exploratory multivariate logistic regression models with at least one statistically significant regression coefficient value predicting the likelihood of a “yes” response to a perception about performing a self-care behavior.

Behavior	Perception of Behavior	Reference	Comparator	β	SE	Wald	df	*p* Value ^a^	OR	95% CI
Seeking more than one expert opinion for the treatment of illness when needed	Able to perform	Rural	Urban	0.898	0.826	1.183	1	0.277	2.455	0.487 to 12.384
		Female	Male	0.657	1.187	0.306	1	0.580	1.929	0.188 to 19.773
		65 to <75 Years				4.344	2	0.114		
			75 to <85 Years	−2.109	1.012	4.341	1	**0.037**	0.121	0.017 to 0.882
			≥85 Years	−1.096	1.199	0.836	1	0.360	0.334	0.032 to 3.502
		Married			0.288	2	0.866			
			Single	−0.478	0.904	0.280	1	0.597	0.620	0.105 to 3.648
			Separated	−0.379	1.272	0.089	1	0.766	0.684	0.057 to 8.279
		<High school			2.019	2	0.364			
			High school diploma	1.157	1.008	1.317	1	0.251	3.180	0.441 to 22.932
			≥Associate’s or bachelor’s degree	−0.060	1.335	0.002	1	0.964	0.942	0.069 to 12.888
Knowing how old and new treatments interact	Able to perform	Rural	Urban	−0.061	0.777	0.006	1	0.937	0.941	0.205 to 4.312
		Female	Male	−0.093	0.975	0.009	1	0.924	0.911	0.135 to 6.160
		65 to <75 Years			6.652	2	0.036			
			75 to <85 Years	−2.422	1.007	5.792	1	**0.016**	0.089	0.012 to 0.638
			≥85 Years	−2.251	1.038	4.699	1	**0.030**	0.105	0.014 to 0.806
		Married			1.907	2	0.385			
			Single	−0.415	0.795	0.273	1	0.601	0.660	0.139 to 3.133
			Separated	−1.800	1.306	1.900	1	0.168	0.165	0.013 to 2.137
		>High school			2.123	2	0.346			
			High school diploma	1.266	0.955	1.758	1	0.185	3.545	0.546 to 23.024
			≥Associate’s or bachelor’s degree	0.396	1.297	0.093	1	0.760	1.486	0.117 to 18.878

^a^ Values in boldface type are statistically significant (<0.05). SE = standard error; df = degrees of freedom; OR = odds ratio; and CI = confidence interval.

**Table 5:** Summary of perceived importance, desirability, and ability to perform 11 patient engagement self-care behaviors for making good treatment decisions and participating in treatment.

Behavior Classification and Analysis Type	Significant Associations ^a^ of Demographics with Perceptions of the Self-Care Behaviors		
	Important to Perform	Desire to Perform	Able to Perform
*Making good treatment decisions (univariate)*			
Seeking more than one expert opinion for the treatment of illness when needed	Compared with adults 65 to less than 75 years of age, adults 85 years of age and older were less likely to perceive this self-care behavior as being important.	—	—
Asking about the good and bad outcomes of suggested treatments	—	—	—
Working with your provider(s) on your treatment plan	—	—	—
*Participating in treatment (univariate)*			
Knowing side effects before starting new treatments	—	—	Compared with older adults having less than a high school education, those with a high school diploma were more likely to report being able to perform this self-care behavior.
Knowing how old and new treatments interact	—	—	Compared with married older adults, separated older adults were less likely to report being able to perform this self-care behavior.
			Compared with adults 65 to less than 75 years of age, adults in the 75 to less than 85 years and the 85 years and older age groups were less likely to report being able to perform this self-care behavior.
Filling or refilling prescriptions on time	—	—	—
Keeping track of the outcomes of your treatments	—	—	—
Talking with your provider(s) when stopping your treatment	—	—	—
Maintaining all of your health devices	—	—	—
Discussing why tests are ordered before getting them done	—	Compared with married older adults, separated older adults were less likely to report desiring to perform this self-care behavior.	—
Tracking your symptoms and health measures	—	—	Compared with adults 65 to less than 75 years of age, adults 85 years of age and older were less likely to report being able to perform this self-care behavior.
*Making good treatment decisions (multivariate)*			
Seeking more than one expert opinion for the treatment of illness when needed	—	—	Compared with adults 65 to less than 75 years of age, adults 75 to less than 85 years of age were less likely to report being able to perform this self-care behavior.
Asking about the good and bad outcomes of suggested treatments	—	—	—
Working with your provider(s) on your treatment plan	—	—	—
*Participating in treatment (exploratory multivariate)*			
Knowing side effects before starting new treatments	—	—	—
Knowing how old and new treatments interact	—	—	Compared with adults 65 to less than 75 years of age, adults in the 75 to less than 85 years and the 85 years and older age groups were less likely to report being able to perform this self-care behavior.
Filling or refilling prescriptions on time	—	—	—
Keeping track of the outcomes of your treatments	—	—	—
Talking with your provider(s) when stopping your treatment	—	—	—
Maintaining all of your health devices	—	—	—
Discussing why tests are ordered before getting them done	—	—	—
Tracking your symptoms and health measures	—	—	—

^a^ In the univariate logistic regression, only one demographic variable was entered into the model. In the multivariate logistic regression, all five demographic variables were entered into the model. Alpha was set to 0.05 for two-sided statistical tests.

## Data Availability

The data used in this study are not open to other researchers at this time.
